# Primary ovarian lymphoma: A case report

**DOI:** 10.1016/j.gore.2023.101212

**Published:** 2023-05-26

**Authors:** Charlotte Gerrity, Alyssa Mercadel, Abrar Alghamdi, Marilyn Huang

**Affiliations:** a Department of Obstetrics and Gynecology, University of Miami, Miami, FL, United States; b Division of Gynecologic Oncology, Sylvester Comprehensive Cancer Center, University of Miami, Miami, FL, United States; c Department of Pathology, University of Miami, Miami, FL, United States

**Keywords:** Ovarian lymphoma, Non-Hodgkin Lymphoma, Ovarian tumors

## Abstract

While Non-Hodgkin Lymphoma (NHL) often involves the ovaries at time of autopsy, it is rarely present at the time of diagnosis. Here we present a case of a 20-year-old who presented with a large adnexal mass and elevated B-HCG, CA-125, and LDH. The patient underwent exploratory laparotomy, and frozen section of the left ovarian mass was suspected to be a dysgerminoma. Final pathologic diagnosis was Ann Arbor stage IVE Diffuse Large B-Cell Lymphoma, germinal center subtype. Patient is currently undergoing chemotherapy and has received the 3 of a planned 6 cycles of R-CHOP.

## Highlights

• Primary ovarian lymphoma is a rare ovarian tumor that should be part of differential diagnosis for adnexal masses.
• Ovarian lymphoma has no distinguishing features on workup.
• Fertility sparing surgery is feasible if lymphoma identified preoperative or intraoperatively.

## 1 Introduction

While ovarian involvement of Non-Hodgkin Lymphoma (NHL) occurs in 7–26 % of cases, it is rarely present at the time of diagnosis. The term ‘primary ovarian lymphoma’ denotes an extranodal lymphoma identified in one or both ovaries without evidence of involvement of any other lymph node region or extra-lymphatic organ. The pathogenesis of ovarian lymphoma is debated due to the lack of lymphatic tissue in the ovary. It is hypothesized to arise from reactive lymphocytic infiltrates in the setting of pelvic inflammatory disease, endometriosis, benign ovarian cysts, or other inflammatory conditions (Crasta and Vallikad, 2009). Primary ovarian lymphoma has been reported in 0.1–0.5 % of female patients with untreated NHL and 1.5 % of patients with an untreated ovarian neoplasm (Dimopoulos et al., 1997). Lymphomas of the ovary present a diagnostic challenge, as on clinical presentation, they may be indistinguishable from a primary gynecologic malignancy (Slonimsky et al., 2018). Here we present a case of a 20-year-old patient with primary NHL of the ovary and review of the literature.

## 2 Case presentation

A 20-year-old healthy woman presented to the emergency department at a tertiary care center with abdominal pain and distension. She reported a growing, firm pelvic mass over the last several months. On abdominal exam, she had a firm, mildly tender pelvic mass extending to the level of the umbilicus. B-HCG was 62.6 mIU/mL on initial presentation, but no intrauterine pregnancy (IUP) or ectopic pregnancy was visualized on transvaginal ultrasound. The pelvic ultrasound did reveal a 14 × 9 × 12 cm left heterogenous adnexal mass with cystic areas and vascularity, and the final radiology report suggested concern for an ovarian dysgerminoma. Tumor markers were collected: CA-125 was elevated to 202 U/mL, CA 19–9 was < 0.6 ng/mL and CEA was 0.9 U/mL. She was referred for outpatient gynecologic oncology follow up.

Three weeks after initial presentation, she returned to the ED with worsening abdominal pain, distension, and lethargy. Beta-HCG had increased to 47,307 mIU/mL. Repeat TVUS demonstrated a viable IUP. The large, heterogenous left adnexal mass was also again visualized. Additional tumor markers were obtained including AFP, LDH, testosterone, and Inhibin B. Testosterone was 55 ng/dl (First trimester reference range 26–211 ng/dl) (Slonimsky et al., 2018) and inhibin B was negative (<10 pg/ml), which decreased suspicion for ovarian sex cord-stromal tumor. LDH was elevated to LDH of 1,714.5 (First trimester reference range 78–433) (Slonimsky et al., 2018) supporting the leading diagnosis of dysgerminoma. AFP was within normal limits. She was admitted for observation and pain control and discharged the following day with plans for follow up with an obstetrics provider for pregnancy care. After extensive counseling, she wished to have an elective termination of pregnancy with mifepristone and misoprostol at 6.6 weeks. A limited in-office ultrasound was performed seven days after medical termination, which demonstrated no IUP and an irregular endometrial lining measuring 20 mm.

At day ten post-termination, the patient presented again to the ED with three days of constant, severe pelvic pain that suddenly worsened the night before presentation. Pain was associated with subjective fever chills, bloating, yellow, non-malodorous vaginal discharge, and constipation. She became febrile to 39.2 degrees Celsius, tachycardic, and had a leukocytosis to 11,200/mcL. On physical exam, her abdomen was diffusely tender to palpation with guarding but no rebound tenderness. Pelvic exam was limited due to the patient’s discomfort. The cervix was unable to be visualized on speculum exam, and bimanual exam demonstrated cervical motion tenderness.

**Fig. 1:**
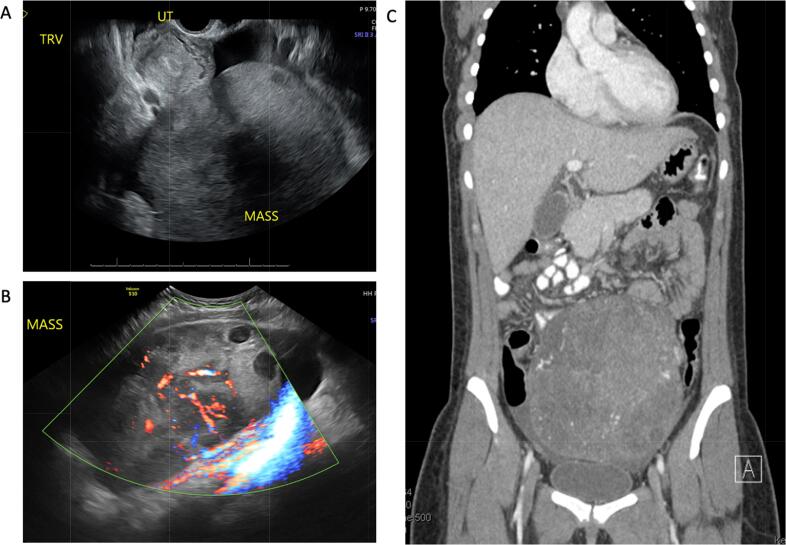
A. Transvaginal ultrasound demonstrating a large, heterogeneous vascular mass in the midline pelvis, heterogeneous echoes within the endometrium, and free fluid in the pelvis. B. Transabdominal ultrasound demonstrating heterogeneous adnexal mass with cystic areas and vascularity. C. CT of the abdomen and pelvis demonstrating 17 cm heterogeneously enhancing pelvic mass, small volume ascites.

Transvaginal ultrasound showed a large, vascular mass in the midline pelvis, an endometrium with vascular, heterogenous echoes measuring 23 mm, and a moderate amount of free fluid in the pelvis (Fig. 1A and 1B). CT of the abdomen and pelvis redemonstrated a 18 cm midline heterogeneously enhancing pelvic mass, enlarged left *para*-aortic lymph nodes measuring up to 1.3 cm, and small volume ascites with mild nodular stranding of the greater omentum (Fig. 1C). CT of the chest did not show evidence of thoracic disease. The patient was admitted to the gynecology service for management of a presumed septic abortion and started on empiric intravenous ampicillin, gentamicin, and clindamycin.

**Fig. 2:**
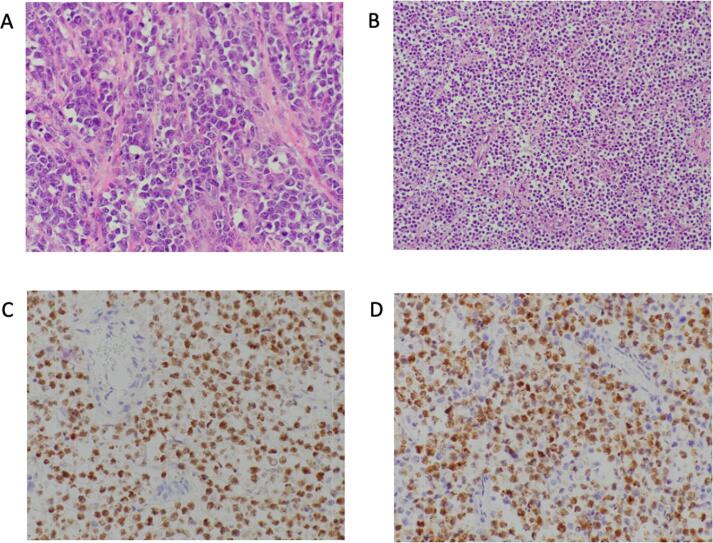
A. High-power view show a diffuse proliferation of large lymphoid cells (B cells) with numerous mitotic figures (Hematoxylin and eosin). B. A low power view of dense infiltrate of lymphoma cells replacing the normal ovarin architecture. (Hematoxylin and eosin). C. Tumor cells are positive for pax-5(B-cell marker). D. Tumor cells are positive for BCL6.

She underwent suction dilation and curettage on hospital day two with significant improvement of pelvic pain and resolution of fevers. Repeat CT of the abdomen and pelvis was performed on postoperative day two. The mass had slightly decreased in size to 16 cm, however there were new hypodense areas suggestive of necrosis. She then underwent exploratory laparotomy, left salpingo-oophorectomy, and omental and peritoneal biopsies. Intraoperative findings were notable for an approximately 15 cm left adnexal mass with no visible normal appearing ovary. On gross examination, the external surface was smooth and gray-tan, and cut sections revealed focal hemorrhage and degeneration. The frozen section was consistent with dysgerminoma. Lymph node sampling was not performed due to low evidence of benefit in ovarian germ cell tumors; thus, nodal sampling was not performed based on frozen section. Final pathology ultimately revealed diffuse large B-cell lymphoma, germinal center subtype involving the left ovary and omentum. Immunohistochemical studies showed that the large lymphoid cells are positive for CD45, CD20, PAX5, BCL6 and C-MYC while negative for CD5, MUM1, BCL-2, and CD138 (Fig. 2D and 2E)). Endometrial pathology revealed an endometrial polyp and no products of conception.

## 3 Discussion

Ovarian lymphoma has variable presentation, and can include symptoms typical of ovarian malignancy such as pelvic pain, bloating, pelvic mass, or ascites. Fevers, night sweats, fatigue, and weight loss (B symptoms) are frequently reported. CT imaging typically demonstrates homogenous masses with uniform enhancement and without cystic components, papillary projections, hemorrhage, necrosis, or calcifications (Slonimsky et al., 2018).

Extranodal NHL is typically diagnosed by incisional or excisional biopsy or fine needle aspiration, and primary treatment typically involves systemic chemotherapy with or without local radiation rather than surgical resection (Shankland et al., 2012). Ovarian malignancies are typically diagnosed and staged surgically, and thus, surgical resection followed by chemotherapy is common for patients with primary ovarian lymphomas, as was the case for this patient (Dimopoulos et al., 1997). It is often observed that patients with primary ovarian lymphomas may experience morbidity from surgical resection that would not typically be performed for an extranodal lymphoma at another site. This morbidity can include delays in initiation of chemotherapy, sequelae of radical surgery such as bowel resection and ostomy creation, and hysterectomy and/or bilateral salpingo-oophorectomy in pre-menopausal patients that may not have a therapeutic benefit (Dimopoulos et al., 1997, Slonimsky et al., 2018, Osataphan et al., 2021, Pectasides et al., 2008).

On initial presentation, the differential diagnosis for the patient was very broad. This patient initially presented with elevated CA-125 and LDH. CA-125 is the most commonly used blood test for ovarian cancer surveillance but has limited use as a screening tool due to unacceptably high false-positive and -negative rates (Gostout and Brewer, 2006). Elevated CA-125 is often reported in patients with ovarian lymphoma, frequently above the threshold where SGO/ACOG recommends referral to a gynecologic oncologist for surgical evaluation of adnexal mass. (Sergi et al., 2021, Sung et al., 2022, Kumar et al., 2014) CA-125 elevation can also be seen in NHL and may be associated with aggressive disease and lower rates of treatment response (Memar et al., 2015). The use of CA-125 as a prognostic marker or for monitoring treatment response in primary ovarian lymphoma has not been described. LDH is also often elevated in ovarian lymphomas (Ambulkar and Nair, 2003). LDH elevation is independently associated with more aggressive disease and worse survival outcomes in NHL regardless of clinical stage (Shankland et al., 2012) LDH is also produced by dysgerminomas, the most common malignant germ cell tumor of the ovary (Mohamed et al., 2022). Positive B-HCG in this case prior to visualization of an intrauterine pregnancy also raised the possibility of embryonal cell carcinoma or ovarian choriocarcinoma.

In this case, CA-125 and LDH elevation, patient age, heterogeneously enhancing pelvic mass on CT, and the finding of malignant neoplasm with small round cells on intraoperative frozen section highly favored the diagnosis of dysgerminoma. Histologically, NHL can resemble dysgerminoma, granulosa cell tumor, small cell neuroendocrine carcinoma, small cell carcinoma of hypercalcemic type, and undifferentiated carcinomas (Vang et al., 2001, Lim et al., 2021). As such, the diagnosis of ovarian NHL is typically not made until immunohistochemical studies can be performed (van Dorp et al., 2013). This has important implications for patients desiring future fertility.

Menstrual recovery and spontaneous pregnancy have been frequently observed in reproductive-aged women receiving chemotherapy for NHL (Elis et al., 2006, Gharwan et al., 2016). Both ovaries are commonly involved in primary ovarian lymphoma, and premenopausal patients with this presentation may undergo bilateral oophorectomy prior to receiving a final pathologic diagnosis of lymphoma (Dimopoulos et al., 1997, Pectasides et al., 2008, Lim et al., 2021). Even when disease is present in both ovaries, fertility-sparing surgical approaches may be feasible if lymphoma is identified on intraoperative frozen section.

## 4 Conclusion

Although primary ovarian lymphoma accounts for a very small proportion of ovarian tumors, it should be a part of a differential diagnosis for adnexal masses suspicious for malignancy. Ovarian lymphoma is difficult to distinguish from a primary ovarian malignancy by imaging, tumor biomarkers, or histology. Maintaining ovarian lymphoma on the differential for an adnexal mass suspicious for malignancy may promote fertility sparing treatment and minimize unnecessary surgical interventions.

## 5 Consents

Written informed consent was obtained from the patient for publication of this case report and all included images. A copy of the written consent is available for review by the Editor-in-Chief of this journal.

## CRediT authorship contribution statement

**Charlotte Gerrity:** Writing – original draft. **Alyssa Mercadel:** Writing – review & editing. **Abrar Alghamdi:** Writing – review & editing. **Marilyn Huang:** Writing – review & editing, Supervision.

## Declaration of Competing Interest

The authors declare that they have no known competing financial interests or personal relationships that could have appeared to influence the work reported in this paper.

## References

[b0005] Ambulkar I., Nair R. (2003). Primary ovarian lymphoma: report of cases and review of literature. Leuk. Lymphoma.

[b0010] Crasta J.A., Vallikad E. (2009). Ovarian LYMPHOMA. Indian J Med Paediatr Oncol..

[b0015] Dimopoulos M.A., Daliani D., Pugh W., Gershenson D., Cabanillas F., Sarris A.H. (1997). Primary ovarian non-Hodgkin's lymphoma: outcome after treatment with combination chemotherapy. Gynecol. Oncol..

[b0020] Elis A., Tevet A., Yerushalmi R., Blickstein D., Bairy O., Dann E.J. (2006). Fertility status among women treated for aggressive non-Hodgkin's lymphoma. Leuk. Lymphoma.

[b0025] Gharwan H., Lai C., Grant C., Dunleavy K., Steinberg S.M., Shovlin M. (2016). Female fertility following dose-adjusted EPOCH-R chemotherapy in primary mediastinal B-cell lymphomas. Leuk. Lymphoma.

[b0030] Gostout B.S., Brewer M.A. (2006). Guidelines for referral of the patient with an adnexal mass. Clin. Obstet. Gynecol..

[b0035] Kumar N., Kumar R., Bera A., Srinivasan R., Sharma S.C. (2014). Primary ovarian lymphoma: a case report and review of literature. J. Obstet. Gynaecol. India.

[b0040] Lim Y.H., Tay A.Z.E., Chew S.H., Aggarwal I. (2021). Primary ovarian lymphoma. Int. J. Gynecol. Cancer.

[b0045] Memar B., Aledavood A., Shahidsales S., Ahadi M., Farzadnia M., Raziee H.R. (2015). The prognostic role of tumor marker CA-125 in B-cell non-Hodgkin's lymphoma. Iran J Cancer Prev..

[b0050] Mohamed A., Ali A., Younis F.M. (2022). Ovarian dysgerminoma. Int. J. Gynecol. Cancer.

[b0055] Osataphan S., Augustynowicz A., Perrino C., Lam P. (2021). Triple-hit high-grade B-cell lymphoma presenting with ovarian torsion. BMJ Case Rep..

[b0060] Pectasides D., Iacovidou I., Psyrri A., Gaglia A., Pectasides E., Papaxoinis G. (2008). Primary ovarian lymphoma: report of two cases and review of the literature. J. Chemother..

[b0065] Sergi W., Marchese T.R.L., Botrugno I., Baglivo A., Spampinato M. (2021). Primary ovarian Burkitt's lymphoma presentation in a young woman: a case report. Int. J. Surg. Case Rep..

[b0070] Shankland K.R., Armitage J.O., Hancock B.W. (2012). Non-Hodgkin lymphoma. Lancet.

[b0075] Slonimsky E., Korach J., Perri T., Davidson T., Apter S., Inbar Y. (2018). Gynecological lymphoma: a case series and review of the literature. J. Comput. Assist. Tomogr..

[b0080] Sung Y.W., Lin Y.S., Chen Y.T., Yeh L.S. (2022). Non-Hodgkin's B-cell lymphoma of the ovary: a case report and review of the literature. Taiwan. J. Obstet. Gynecol..

[b0085] van Dorp W., Owusuaa C., Laven J.S., van den Heuvel-Eibrink M.M., Beishuizen A. (2013). Characteristics and outcome of pediatric non-Hodgkin lymphoma patients with ovarian infiltration at presentation. Pediatr. Blood Cancer.

[b0090] Vang R., Medeiros L.J., Warnke R.A., Higgins J.P., Deavers M.T. (2001). Ovarian non-Hodgkin's lymphoma: a clinicopathologic study of eight primary cases. Mod. Pathol..

